# Children's perceptions of parental emotional neglect and control and psychopathology

**DOI:** 10.1111/j.1469-7610.2011.02390.x

**Published:** 2011-03-25

**Authors:** Robert Young, Susan Lennie, Helen Minnis

**Affiliations:** 1 MRC Social & Public Health Sciences UnitGlasgow, UK; 2 Department of Psychological Medicine, University of GlasgowUK

**Keywords:** DSM, emotional abuse, parent–child relationships, perception, longitudinal studies

## Abstract

**Background:**

Parental emotional neglect is linked to psychiatric disorder. This study explores the associations between children's perceptions of parental emotional neglect and future psychopathology.

**Methods:**

In a school-based longitudinal study of nearly 1,700 children aged 11–15 we explored children's perceptions of parenting, as measured by the Parental Bonding Instrument (PBI) at age 11, and their associations with later psychiatric diagnosis at age 15, as measured by computerised psychiatric interview. Rather than using the traditional four-category approach to the PBI, we identified groups of children, classified according to their perceptions of parenting, using latent class analysis.

**Results:**

A small group of children (3%) perceived their parents as almost always emotionally neglectful and controlling. This group had an increased odds of psychiatric disorder (OR 2.14; 95% CI 1.29–4.50), increased overall (standardised) psychiatric symptom scores (B = .46; 95% CI .16–.75) and increased scores in all psychiatric subscales except substance-use at age 15, despite no increase in psychiatric referral at age 11. Analyses controlled for key potential confounders (e.g., socioeconomic status).

**Conclusions:**

Although our findings are limited by having no objective evidence that children's perceptions of emotional neglect are directly associated with actual neglect, children's perceptions of neglect and control are associated with over twice the odds of psychiatric disorder at age 15. Children's perceptions that parents are emotionally neglectful and controlling are independently associated with later psychiatric disorder and should be taken seriously as a risk factor for future psychopathology.

Emotional neglect (EN) is a major risk factor for psychopathology, including internalising problems such as depression and anxiety (Colvert et al., 2008) and externalising problems including violence (Chapple, Tyler, & Bersani, 2005). Terminology is confusing (APSAC, 1995; Egeland, 2009; Glaser, 2002): when referring to EN we mean ‘emotional unresponsiveness, unavailability and neglect characterised by lack of interaction between parent and child’ (Glaser, 2002). EN and abuse commonly co-occur, but the effects differ (Lee & Hoaken, 2007): compared to physically abused children, neglected children have more severe cognitive and academic deficits, are more socially withdrawn, have limited peer interactions and more internalising (as opposed to externalising) problems. Retrospective evidence suggests that EN is more strongly associated with psychological symptoms than physical abuse (Gauthier, Stollack, Messe, & Arnoff, 1996) and prospective data suggests that EN is associated with personality disorder in adolescence and adulthood (Johnson, Smailes, Cohen, Brown, & Bernstein, 2000).

Developmental trajectories from EN to psychopathology in adolescence and adulthood are still poorly understood (Glaser, 2000; Hildyard & Wolfe, 2002), with complex interactions between genetics and environment (Rutter, Kim-Cohen, & Maughan, 2006). The first years of life mark the period of most rapid change in the human brain (Huttenlocher & Dabholkar, 1997; Teicher et al., 2003, 2004) and this is when the child is most vulnerable to the effects of EN (Hildyard & Wolfe, 2002), but a child exposed to EN in infancy may also be vulnerable to its effects later in childhood. Lack of emotional interaction during the crucial early period of development can result in poor emotional regulation (Lee & Hoaken, 2007; Teicher et al., 2004) that may be part of a cascade of adverse neurobiological events rendering a child vulnerable to the effects of continuing EN as childhood progresses (Teicher et al., 2004).

A young person's ability to integrate information from the environment, both cognitively and emotionally, influences neurobiological development (Lee & Hoaken, 2007). Cognitive attributional biases can result from physical abuse, leading to aggression (Dodge, Bates, & Pettit, 1990): the way children process their own early-childhood experience of violence has an important impact on the way they perceive future social situations. Such biases affect the way children behave in social situations, hence influencing what actually happens (Dodge et al., 1990). There is little comparable research regarding neglect; however, there is some evidence that neglected children may have difficulty discriminating emotional expression (Fries & Pollak, 2004). Neglected children have various attentional and social deficits (Chugani et al., 2001; Turgeon & Nolin, 2004) and it may be that the *perception* of parental neglect (which may or may not stem from *actual* parental neglect) can influence both the child's future experience of social situations and the actuality of those situations. This could result in a vicious cycle towards psychopathology. There is some evidence to support this hypothesis: in a questionnaire study of college students, those who recalled EN were more likely to report maladaptive schemas of vulnerability to harm, shame, and self-sacrifice (O'Dougherty Wright, Crawford, & Del Castillo, 2009).

The focus has so far centred on various retrospective studies of adults’ perceptions of the parenting they received during childhood and associations with concurrent psychopathology, including depression (Yoshida, Taga, Matsumoto, & Fukui, 2005), borderline personality disorder (Nickell, Waudby, & Trull, 2002), eating disorders (Hedlund, Fichter, Quadflieg, & Brandi, 2003) and conduct disorder (Mak, 1994). Many of these studies have used the Parental Bonding Instrument (PBI), a well-validated questionnaire which explores parenting experienced in childhood, traditionally in the domains of parental care and control. From the PBI a ‘parenting typology’ is sometimes created by assigning perceived parenting to one of four quadrants using care and control scores. These four parental rearing styles have been labelled as ‘optimal bonding’ (high care – low control); ‘neglectful parenting’ (low care – low control); ‘affectionate constraint’ (high care – high control) and ‘affectionless control’ (low care – high control), illustrated in Figure 1. It is assumed that certain rearing styles, such as ‘affectionless control’, are risk factors for developing psychiatric conditions. Although a number of groups have now investigated psychopathology in mid to late adolescence and perceptions of parental care/control during childhood using the PBI (Chambers, Power, Loucks, & Swanson, 2001; Patton, Coffey, Posterino, Carlin, & Wolfe, 2001), there is a dearth of prospective research (Yates & Wekerle, 2009) and to our knowledge no study has examined perceptions of parental care/neglect during middle childhood as a *predictor* of later psychopathology.

**Figure 1:**
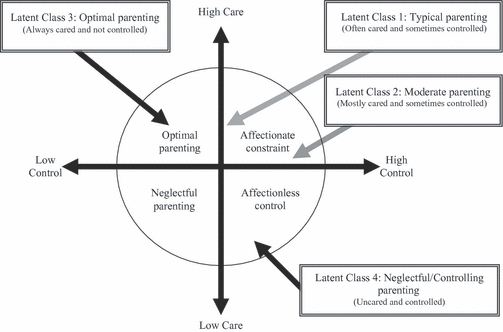
Relationship between conventional ‘parental bonding’ parental styles and styles derived through latent class analysis. Note: Solid arrows indicate congruence between latent classes and conventional PBI parental styles and those derived from LCA, while shaded arrows indicate a lack of congruence

In contrast to past research we have taken an empirical approach and have used latent class analysis (LCA) to investigate the structure of the PBI within the dataset. This means that we focus on the respondent's perspective rather than imposing any predefined position.

We set out to determine whether there were associations between perceptions of parental neglect during childhood and future psychopathology in a longitudinal study of 1,694 young people first surveyed at age 11 and again at age 15.

## Methods

### Sample

Participants were from a longitudinal school-based survey of health and lifestyles in a cohort of young people resident in the West of Scotland. They were first surveyed at the age of 11 years in their final year of primary school (in 1994) and followed up in secondary schools at the ages of 13 (1996) and 15 years (1999). All children in mainstream education in the study area (*n*=2,793) were eligible for the study. Children not in mainstream education (<1% at primary school and 3% at secondary school) were excluded and because excluded children are likely to disproportionately include children with psychiatric problems, the prevalence of psychiatric disorder is likely to be underestimated in the sample. At the age of 11 years, 2,586 (93% of those eligible) children participated. Of the 207 not taking part, most (*n*=181) were withdrawn at the request of parents with the remainder being absentees. At age 15, 2,196 (79%) of the original eligible population took part. Non-participants were mainly absentees and included long-term truants. At age 11, 2,237 (87%) of participating children's parents completed a parental questionnaire. At age 15, 1,860 (67%) of respondents completed a psychiatric interview. For the psychiatric interview, additional positive consent was sought from pupils and few (*n*=48) declined to take part. After excluding those with missing data in other variables, 1,694 (1,667 weighted) cases were available for analysis. The study was approved by the non-clinical research ethics committee at the University of Glasgow.

### Measures

The PBI is a self administered questionnaire that is based on the assumption that parental rearing can be measured by two dimensions of parental care and parental control. The items are scored on a 4-point Likert scale with approximately half of the scale items referring to parental control and half to parental care. Various studies have demonstrated acceptable test–retest reliability (Favaretto, Torresani, & Zimmermann, 2001). Parental rearing styles as measured by the PBI can be assigned to one of four quadrants: ‘optimal bonding’ (high care – low control); ‘neglectful parenting’ (low care – low control); ‘affectionate constraint’ (high care – high control) and ‘affectionless control’ (low care – high control), illustrated in Figure 1. The PBI has both short and long versions, which both correlate well with more direct questionnaire measures of recall of childhood maltreatment (Lancaster, Rollinson, & Hill, 2007). It has been shown to be stable, over a 20-year period, in adults (Wilhelm, Niven, Parker, & Hadzi-Pavlovic, 2004). In this study we used a short 8-item version, the PBI-BC, developed by Klimidis and colleagues, which aims to measure *current* perceptions of parenting in adolescents. It has a similar factor structure to the full PBI which includes two dimensions referring to parental care (e.g., parental love, help, understanding) and (over)control (e.g., treated like a baby) (Klimidis, Minas, & Alta, 1992). Table 1 shows the PBI-BC items.

**Table 1:** Probabilities and proportions for 4-class latent class solution for age 11 sample

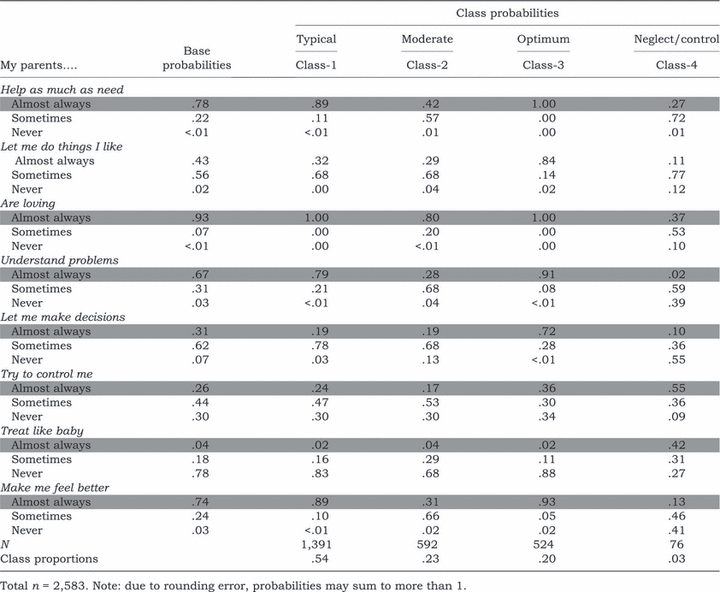

Additional indicators of parent–child interactions and family conflict were measured. At age 11 children reported how well they ‘get on with’ their maternal and paternal figure on a 3-point (not so well, quite well, or very well) scale. An index of family activity was constructed from the average frequency (5-point scale, never to everyday) of six shared family activities: watch TV, play indoors, family meal, walk or play sport, go places, visit friends or relatives. Arguments with parents were measured on the same frequency scale and responses dichotomised into argue most days or everyday vs. argue less often. Parents were asked identical questions about shared family activities and a ‘parental’ family activity index constructed. A family arguments index was constructed using the average frequency (same 5-point scale) for five common sources of arguments: money, tidiness, homework, friends, and helping around the house. See Table 2 for descriptive statistics.

**Table 2:** Descriptive statistics of variables and validation against reported family activity and arguments

	Optimum parenting *n*=325, 19.8%		Typical parenting *n*=923, 56.3%		Moderate parenting *n*=342, 20.8%		Neglectful and controlling parenting *n*=49, 3.0%		
					
Variables*	*n*	%	*n*	%	*n*	%	*n*	%	χ^2^ or *F-test*
Demographics									
Gender									
Male (*n*=803, 48.2%)	161	49.5	450	48.8	159	46.6	20	40.0	.566
Social class									
Missing (dummy variable)	16	4.9	61	6.6	21	6.1	7	14.3	
Non-manual	129	39.8	404	43.8	131	38.3	17	34.7	
Manual	179	55.2	458	49.6	190	55.6	25	51.0	.083
Area deprivation category									
Missing	48	14.8	118	12.8	67	19.6	13	26.5	
1	31	9.6	82	8.9	35	10.3	3	6.1	
2	24	7.4	79	8.5	23	6.7	2	4.1	
3	50	15.4	138	14.9	37	10.9	4	8.2	
4	46	14.2	114	12.3	49	14.4	7	14.3	
5	45	13.9	136	14.7	31	9.1	5	10.2	
6	38	11.7	113	12.2	47	13.8	6	12.2	
7	42	13.0	144	15.6	52	15.2	9	18.4	.095
Family structure at age 11 [MD = 16]									
2-parent	248	76.3	702	76.0	245	71.8	35	70.0	
1-parent, + other (reconstituted)	25	7.7	81	8.8	41	12.0	7	14.0	
1-parent	52	16.0	141	15.3	55	16.1	8	16.0	.422
Child report									
Get on with mum/step-mum [MD = 22]									
Not so well (vs very/quite well)	0	.0	2	.2	6	1.8	8	17.8	≤.001
Get on with dad/step-dad [MD = 18]									
Not so well (vs very/quite well)	0	.0	8	1.0	7	2.5	16	36.4	≤.001
Argue most days with parents	44	18.2	165	21.6	101	34.0	33	71.7	≤.001
Family activity score † (*M*, *SD*)	3.46	.62	3.34	.62	3.04	.59	2.75	.80	≤.001
Depression & anxiety score, age 11 (*M*, *SD*)	14.70	3.46	15.60	3.48	16.06	3.45	17.37	3.60	≤.001
Parent report at age 11									
Family activity score † (*M*, *SD*) [MD = 239]	3.36	.45	3.36	.45	3.32	.50	3.42	.57	.502
Argue score † (M, SD) [MD = 240]	2.19	.70	2.22	.70	2.29	.70	2.75	.79	≤.001
Past service contact (before age 11)									
Social services [MD = 241]	0	.0	19	2.3	6	1.9	1	2.6	.084
Psychology/psychiatry [MD = 242]	9	3.2	38	4.8	11	3.6	0	.0	.348

* Weighted data reported, *n*=1,667 used (excluding missing cases). Due to weighting, totals may be more or less than 1,667. MD = Missing data.

† = 5-point scale (everyday to never).

### DSM-IV diagnosis and symptoms

A computerised version of the Diagnostic Interview Schedule for Children (DISC) – the Voice-DISC – was used to collect psychiatric data (Lucas, 2003). The choice of the Voice-DISC was influenced by study design, our prior experience with the instrument and its capacity to be administrated in a school setting (West, Sweeting, Barton, & Lucas, 2003). It is a replica of the interviewer version of the DISC and equally reliable (Lucas, 2003). Respondents self-administer the interview, using a laptop computer. Following an introduction, the Voice-DISC interview proceeds through a series of sections. Questions are asked to establish the presence of symptoms, their severity and duration, and the extent to which they cause distress and/or impairment. Disorders included in this study comprise four anxiety disorders (social phobia, panic, generalised anxiety disorder, and obsessive-compulsive disorder), two depressive disorders (major depressive and dysthymia), eating disorders, and three externalising (ADHD, ODD and CD) disorders and alcohol/substance abuse and dependence. Several disorders were excluded: e.g., schizophrenia on the grounds of inappropriateness.

Voice-DISC produces present-state (previous 4 weeks) diagnoses in accordance with DSM-IV criteria (American Psychiatric Association, 1994) and we focus exclusively on DSM-IV diagnoses based on symptom criteria. In addition to specific diagnoses, the Voice-DISC produces a symptom count for several disorders.

### Social and demographic controls

Several social background measures at age 11 were included in our analysis as control variables, including gender. An area deprivation score, range 1 (least) to 7 (most deprived), was derived from pupils’ postal codes using the ‘Carstairs’ (McLoone, 2004) index, a standard measure based upon census data. Household socioeconomic status (head of household) was derived from parental questionnaires (age 11), coded using the standard UK classification system (ONS, 2000) and categorised as non-manual, manual, or missing. Family structure was coded as 2-parent, 1-parent, reconstituted (one ‘birth’ parent and new partner) or other (relative, foster parent, or other carer). To assess previous psychiatric and social problems, parents were asked about past contact with social and psychiatric services since age 11. Levels of depression and anxiety at age 11 were assessed using the Kandel and Davies Depression Scale (Kandel & Davies, 1982). See Table 2 for descriptive statistics.

### Data analysis

We conducted latent class analysis on the 8-item PBI-BC. Using standard fit criteria, the Lo–Mendell–Rubin's Likelihood Ratio Test and substantive interpretation of the results, a four-class solution was selected as the ‘best fit’ and produced four broadly similar classes across all three waves (full results available from RY). We used logistic and linear regression to determine the association between broad diagnostic categories of psychiatric disorders and symptoms at age 15 and perceived parental rearing at age 11. Weights to compensate for differential attrition were constructed, although results using weights were substantively no different from unweighted results. In a previous analysis weighting increased the prevalence of conduct disorder; accordingly, we report only weighted findings.

## Results

The latent class analysis of the PBI-BC at age 11, age 13 and age 15 suggested a four-class solution, but owing to space limitations only the age 11 results are summarised in Table 1. For a sample of 2,583 children with complete PBI data, only a small (3%, *n*=76) group of children at age 11 perceived their parents as ‘neglectful and controlling’. Focusing on the ‘almost always’ category (highlighted for clarity), of the four groups neglected and controlled children perceived themselves to be helped least, least likely to be allowed to do things they like, least loved, least understood, least likely to be allowed to make decisions, most controlled, most often treated like a baby and least likely to be made to feel better by their parents. Approximately 20% were categorised as the ‘optimal parenting’ group. Again focusing on the ‘almost always’ category, these children perceived themselves as the most helped, most often allowed to do things they like, most loved (tied with the typical group) most understood, most allowed to make decisions, second most controlled, least likely to be treated like a baby (tied with the typical group) and most likely to be made to feel better by their parents. There was a large (approximately 54%) ‘typical parenting’ group. Restricting our attention to the ‘almost always’ category, these children perceived themselves as the second most helped, second most often allowed to do things they like, most loved (tied with the optimum group), second most understood, second most allowed to make decisions (tied with the moderate group), second most controlled, third most likely to be treated like a baby (tied with the optimum group) and second most likely to be made to feel better by their parents. Finally there was a ‘somewhat tougher and stricter parenting’ (23%) group (referred to as the ‘moderate’ parenting group henceforth). Looking at the ‘almost always’ category, these children perceived themselves as the third most helped, third most often allowed to do things they like, third most loved, third most understood, third most allowed to make decisions (tied with the typical group), the fourth most controlled, second most likely to be treated like a baby and third most likely to be made to feel better by their parents.

While we find convergence in our latent class analysis between ‘optimal parenting’ and our ‘optimum’ latent class, and between ‘affectionless control’ and our ‘neglectful and controlling’ latent class, we found no evidence for the existence of either a purely ‘neglectful parenting’ class in which children experienced low control or an ‘affectionate constraint’ group, see Figure 1. In contrast, we found the majority of cases belonged to either a ‘typical parenting’ (moderate care – lower control) or a ‘moderate parenting’ (moderate care – moderate control) group.

We explored the associations between latent class groups and our measures of parent–child interactions in order to validate the classes. Generally, at age 11 our latent class groups are unrelated to demographic factors such as gender, social class, area deprivation or family structure, nor are they associated with contact with psychiatric/psychological or social services (Table 2). At age 11, children in the neglect and control group reported that they did not ‘get on so well’ with either parent, argued most days with parents and engaged in fewer family activities. Parents of children in the neglect and control group also reported more frequent family arguments. In contrast to contact with psychiatric/social services, each group experienced significantly different levels of depression, with progressively higher depression scores for optimal, typical, moderate and neglect/control groups respectively.

Compared to the ‘optimal’ group, the ‘neglectful and controlling parenting’ group had a more than twofold increase in the odds of a psychiatric disorder at age 15 (Table 3). There was also a modest increase in odds of any disorder in the ‘typical’ group (OR 1.33, *p* = .051), but this was small in comparison to the neglected and controlled group (OR 1.33 vs. 2.41). There were no significant gender interactions, but amongst females, the neglected and controlled group was nearly 6 times more likely (OR 5.96; 95% CI .98–36.48; *p* = .055) to suffer from a depressive disorder than the optimum group.

**Table 3:** Associations (odds ratio) between PBI latent class and major psychiatric disorder, adjusted for social background

		Gender	Adjusted^1^ association with perceived parenting OR (95% CI)			
			
Diagnosis	Rate % (*n*)	Interaction (*p*-level)	Optimum	Typical	Moderate	Neglectful and controlling
Anxiety disorder	9.2 (153)	.612	Ref	1.10 (.71–1.73) *p* = .658	1.10 (.65–1.90) *p* = .703	1.37 (.53–3.70) *p* = .494
Depressive disorder	2.3 (38)	n/a 2	Ref	1.40 (.54–3.61) *p* = .491	1.64 (.55–4.84) *p* = .371	3.10 (.60–16.04) *p* = .177
Behavioral disorder (inc. ADHD) 3	12.3 (201)	.916	Ref	1.40 (.91–2.15) *p* = .130	1.30 (.79–2.15) *p* = .303	2.07 (.93–4.65) *p* = .076
Substance abuse or dependence	19.3 (323)	.118	Ref	1.17 (.84–1.65) *p* = .351	1.23 (.83–1.84) *p* = .298	2.13 (1.06–4.26) *p* = .033
Any disorder	30.8 (513)	.243	Ref	1.33 (1.00–1.78) *p* = .051	1.22 (.86–1.72) *p* = .259	2.41 (1.29–4.50) *p* = .006

Ref = reference group.

1 Adjusted for gender, area deprivation, social class and family structure.

2 Test omitted due to low numbers.

3 ADHD combined with ODD and CD because of low rates for ADHD and the similarity of results (see Table 4 for separate symptom scores results).

With respect to standardised psychiatric symptoms there were modest increases in symptoms in the typical and moderate parenting groups, compared to the optimum parenting group for all domains of psychopathology except substance disorder symptoms (Table 4). However, the neglected and controlled group (with the exception of substance disorder) had significantly higher symptom scores in all domains at age 15.

**Table 4:** Associations (standard regression) between PBI latent class and psychiatric symptoms, adjusted for social background

	Gender interaction	Adjusted^1^ association with perceived parenting B (95% CI)			
		
Symptoms scores (standardised *z*-score)	*Δ*F (*p*-level)	Optimum	Typical	Moderate	Neglectful and controlling
Anxiety scores (Avg social phobia, GAD & OCD)	.030	Ref	.20 (.07–.32) *p* = .002	.20 (.05–.35) *p* = .008	.43 (.14–.73) *p* = .004
Mood disorder scores (MMD & Dysthymic symptoms)	.424	Ref	.21 (.08–.33) *p* = .001	.30 (.15–.45) *p* ≤ .001	.31 (.02–.61) *p* = .036
Conduct problem scores (Avg CD & ODD)	.075	Ref	.23 (.11–.35) *p* ≤ .001	.23 (.08–.38) *p* = .002	.41 (.12–.70) *p* = .006
ADHD scores	.587	Ref	.26 (.14–.39) *p* ≤ .001	.35 (.20–.50) *p* ≤ .001	.30 (.00–.59) *p* = .050
Substance-use scores (Avg alcohol, marij, nicotine, other)	.758	Ref	.03 (–.10–.15) *p* = .684	.07 (–.08–.23) *p* = .337	.13 (–.17–.42) *p* = .410
Total score (Avg of all symptoms)	.163	Ref	.24 (.11–.36) *p* ≤ .001	.29 (.14–.44) *p* ≤ .001	.46 (.16–.75) *p* = .003

Ref = reference group.

1 Adjusted for gender, area deprivation, social class and family structure.

There were gender differences for symptoms: among males, all but optimum parenting was associated with increased anxiety symptoms (B = .33, 95% CI .16–.50, *p* < .001; B = .23, CI .03–.43, *p* = .025; B = .33, CI −.04–.70, *p* = .084, for typical, moderate and neglectful/controlling parenting groups respectively). Among females, however, only the neglected and controlling group had significantly increased anxiety symptoms (B = .59, 95% CI .11–1.07, *p* = .016). Among females all but optimal parenting was also associated with an increase in conduct disorder symptoms (B = .14, 95% CI −.02–.29, *p* = .086; B = .29, CI .10–.48, *p* = .003; B = .14, CI −.27–.55, *p* = .502, for typical, moderate and neglectful/controlling parenting respectively). Among males all but optimal parenting was associated with an increase in conduct disorder symptoms, but the neglected and controlled group displayed particularly high symptoms (B = .30, 95% CI .11–.49, *p* = .002; B = .17, CI −.05–.40, *p* = .138; B = .61, CI .19–1.03, *p* = .004 for typical, moderate and neglectful/controlling parenting respectively).

The entire set of analyses was repeated, adjusting for age 11 levels of depression symptoms (see Web appendices 1 and 2). As expected, this attenuated the significance levels; however, including these in the regression analyses did not substantively alter the effect size of age 15 psychiatric outcomes. For example, unadjusted for prior depression the neglected and controlled group show a significant increase in the odds of receiving diagnosis when compared to the optimal group (OR 2.41, *p* = .006) and while the *p*-levels are attenuated the adjusted effect size is similar (OR 2.05, *p* = .026). This was also broadly true of the analyses using the standardised symptom scores. For example, unadjusted the neglected and controlled group show significantly higher symptom scores than the optimal group (B = .46, *p* = .003); the equivalent adjusted effect size is (B = .28, *p* = .055).

## Discussion

Generally speaking, only young people who perceived parental neglect and control show an increased risk of developing psychiatric *disorders*; however, with the exception of substance-use, young people who perceived all but optimal parenting show increased psychiatric *symptoms*. This is compatible with the conventional psychiatric vulnerability/threshold model (American Psychiatric Association, 1994). It is striking that for 97% of the children in the sample, there is very little association between perceived parenting at age 11 and psychopathology (in terms of a DSM-IV diagnosis), despite there being quite a wide range of perceptions of parenting quality within this group. This resonates with Winnicott's notion of ‘good enough parenting’ (Winnicott, 1965). Nonetheless, our crudest DSM-IV diagnosis (any diagnosis) suggested that children perceiving less than optimal parenting report greater levels of psychopathology. Our results for symptom scores are also compatible with this suggestion, since increases in symptom scores were associated with all types of parenting except the optimum and reinforce the notion that sub-optimal parenting has detrimental effects on psychological development (Prevatt, 2003), even if not leading to psychiatric diagnosis.

A small, but significant, percentage of children perceived their parents as being very unloving yet controlling at age 11 and this group of children are more than twice as likely to report a psychiatric disorder at age 15. This constellation of perceived neglect and control, sometimes referred to as ‘affectionless control’, has already been shown to be associated, in cross-sectional studies, with psychopathology (Nickell et al., 2002; Yoshida et al., 2005), but the direction of causality has never been clear. In this study, perception of parental neglect and control precedes the onset of a psychiatric disorder. Supplementary analyses (Web appendices 1 and 2) of depression and anxiety symptom scores (Kandel & Davies, 1982), measured at age 11, demonstrated that, although children who perceive parental neglect and control had elevated symptoms of psychopathology, including these in the regression analysis had little impact on the effect size of age 15 psychiatric outcomes. In addition, none of the children who perceived their parents as neglectful and controlling at age 11 were involved with psychiatric services at that age. Paradoxically, lack of service contact could be interpreted as further validation of neglect.

Classifying emotional neglect and abuse is difficult (Glaser, 2002) because these risks often co-occur. Looking at the items endorsed by the small neglected and controlled (i.e., low care – high control) group, it is clear that the overwhelming experience of these children and young people is of being ignored and failing to have their needs met by their parents – but also of being controlled. Previous research has shown that young children whose needs are not met tend to become more angry and less compliant later in childhood (Egeland, Sroufe, & Erickson, 1983) and it may be that a parent who already has poor interaction with their child is likely to become controlling as a mechanism for managing this behaviour. It is important to explore the relationship between traditional PBI categories and naturally occurring classifications. One advantage of using latent class analysis is that we are not allocating children to groups on an arbitrary basis. We take a respondent-driven rather than researcher-imposed approach and empirically explore what proportion of the child population can be allocated to distinct groups within this sample. The groups revealed by our latent class analysis only partially map onto the traditional four categories used in previous parental bonding research (see Figure 1). While we find convergence in our latent class analysis between ‘optimal parenting’ and our ‘optimum’ latent class, and between ‘affectionless control’ and our ‘neglected/controlled’ latent class, we found no evidence for the existence of either a distinct ‘neglectful parenting’ or an ‘affectionate constraint’ group. It is an important task of future research to establish whether our empirically derived categories can be replicated in other general population and clinical samples.

Our findings are limited by some sample attrition. Although this has been addressed to some extent by the use of weighting, it is likely that we have differentially lost participants at greater risk of suffering from psychiatric disorders, since psychiatric disorder is known to be linked to truancy and absenteeism (Egger, Costello, & Angold, 2003). This, combined with the exclusion of specialist schools in the study, is likely to have affected the precision of our results. Because we did not measure psychopathology at age 11, it is possible that children who perceived their parents as neglectful and controlling were already affected by psychiatric disorders and that these disorders affected their perceptions. While this is unlikely (because none of this group were in contact with psychiatric services at this age), we cannot exclude the possibility that at age 11 a pre-existing psychiatric disorder may have been overlooked by parents, teachers and others with a duty of care. It is impossible to be certain, in this study, whether it is children's *perceptions* of neglect and control that account for the increased prevalence of psychiatric disorder or whether perception of neglect and control is a proxy for *actual* neglect. Future research incorporating data linkage with data from child protection services could address this.

The strong association between children's perceptions of parental neglect and control and psychopathology was confounded very minimally by important indices such as family structure. This suggests that perception of neglect is an important factor in its own right, rather than just a proxy for other indices of adversity or social class. This links well with recent research on the importance of shared meaning, attunement or intersubjectivity between carer and child. Animal and human research has shown that, particularly in infancy, the young organism is programmed to ‘tune in’ to caregivers (Trevarthen & Aitken, 2001). In our study, children categorised as neglected and controlled reported being routinely ignored and unsupported by parents and therefore may lack crucial daily experiences that provide scaffolding for healthy development.

These findings emphasise the importance of eliciting children's perceptions of parenting as part of routine clinical assessment in child and adolescent mental health services. Whether or not children provide accurate accounts is probably less relevant than the perceptions themselves and these should be taken seriously as an indicator of risk for future psychopathology (Jensen et al., 1999). Our findings also have implications for prevention at both the clinical and population level: from a clinical perspective it suggests that children's perceptions of neglect and control precede the development of psychiatric disorders, and from a public health perspective it suggests a link between perceptions of parenting and more general mental health. It is therefore important that professionals working with children, such as teachers, youth workers and social workers, do not trivialise the importance of children's perceptions of parenting: if a child complains that a parent is never loving, understanding or supportive, this may be a powerful indicator with important implications for future mental health.

**Key points**

- Emotional neglect by parents is linked to many types of childhood and adult psychiatric disorders and symptoms.
- Large, representative, prospective studies of perceived emotional neglect and later psychopathology are rare.
- Using a prospective design we found that emotional neglect and control at age 11 significantly predicted psychopathology at age 15. However, only extreme perceived emotional neglect was associated with later psychiatric diagnosis, while less than optimal parenting predicted elevated levels of psychiatric symptoms.

## Correspondence to

Robert Young, MRC SPHSU, 4 Lilybank Gardens, Glasgow G12 8RZ, UK; Email: robert@sphsu.mrc.ac.uk

## Supporting information

**Web appendix 1.** Associations (odds ratio) between PBI latent class and major psychiatric disorder, adjusted for social background and age-11 depression score (Word document).

**Web appendix 2.** Associations (standard regression) between PBI latent class and psychiatric symptoms, adjusted for social background and age-11 depression score (Word document).

## References

[b1] American Psychiatric Association (1994). Diagnostic and statistical manual of mental disorders (DSM-IV).

[b2] American Professional Society on the Abuse of Children (APSAC) (1995). Guidelines for the psychosocial evaluation of suspected psychological maltreatment in children and adolescents.

[b3] Chambers J, Power K, Loucks N, Swanson V (2001). The interaction of perceived maternal and paternal styles and their relation with the psychological distress and offending characteristics of incarcerated young offenders. Journal of Adolescence.

[b4] Chapple CL, Tyler KA, Bersani B (2005). Child neglect and adolescent violence: Examining the effects of self-control and peer rejection. Violence and Victims.

[b5] Chugani HT, Behen ME, Muzik O (2001). Local brain functional activity following early deprivation: A study of postinstitutionalized Romanian orphans. NeuroImage.

[b6] Colvert E, Rutter M, Beckett C, Castle J, Groothues C, Hawkins A (2008). Emotional difficulties in early adolescence following severe early deprivation: Findings from the English and Romanian adoptees study. Development and Psychopathology.

[b7] Dodge KA, Bates JE, Pettit GS (1990). Mechanisms in the cycle of violence. Science.

[b8] Egeland B (2009). Taking stock: Childhood emotional maltreatment and developmental psychopathology. Child Abuse and Neglect.

[b9] Egeland B, Sroufe A, Erickson MT (1983). The developmental consequences of different patterns of maltreatment. International Journal of Child Abuse and Neglect.

[b10] Egger H, Costello AJ, Angold A (2003). School refusal and psychiatric disorders: A community study. Journal of the American Academy of Child and Adolescent Psychiatry.

[b11] Favaretto E, Torresani S, Zimmermann C (2001). Further results on the reliability of the Parental Bonding Instrument (PBI) in an Italian sample of schizophrenic patients and their parents. Journal of Clinical Psychology.

[b12] Fries AB, Pollak SD (2004). Emotion understanding in postinstitutional Eastern European children. Development and Psychopathology.

[b13] Gauthier L, Stollack G, Messe L, Arnoff J (1996). Recall of childhood neglect and physical abuse as differential predictors of current psychological functioning. Child Abuse and Neglect.

[b14] Glaser D (2000). Child abuse and neglect and the brain – a review. Journal of Child Psychology and Psychiatry.

[b15] Glaser D (2002). Emotional abuse and neglect (psychological maltreatment): A conceptual framework. Child Abuse and Neglect.

[b16] Hedlund S, Fichter MM, Quadflieg N, Brandi C (2003). Expressed emotion, family environment and parental bonding in bulimia nervosa: A 6 year investigation. Eating and Weight Disorders.

[b17] Hildyard KL, Wolfe DA (2002). Child neglect: Developmental issues and outcomes. Child Abuse and Neglect.

[b18] Huttenlocher PR, Dabholkar AS (1997). Regional differences in synaptogenesis in human cerebral cortex. Journal of Comparative Neurology.

[b19] Jensen P, Rubio-Stipec M, Canino G, Bird H, Dulcan M, Schwab-Stone M (1999). Parent and child contributions to diagnosis of mental disorder: Are both informants always necessary?. Journal of the American Academy of Child and Adolescent Psychiatry.

[b20] Johnson JG, Smailes EM, Cohen P, Brown J, Bernstein DP (2000). Associations between four types of childhood neglect and personality disorder symptoms during adolescence and early adulthood: Findings of a community-based longitudinal study. Journal of Personality Disorders.

[b21] Kandel DB, Davies M (1982). Epidemiology of depressive mood in adolescents: An empirical study. Archives of General Psychiatry.

[b22] Klimidis S, Minas I, Alta A (1992). The PBI-BC: A brief form of the parental bonding instrument for adolescent research. Comprehensive Psychiatry.

[b23] Lancaster G, Rollinson L, Hill J (2007). The measurement of a major childhood risk for depression: Comparison of the Parenting Bonding Instrument (PBI) ‘Parental Care’ and the Childhood Experience of Care and Abuse (CECA) ‘Parental Neglect’. Journal of Affective Disorders.

[b24] Lee V, Hoaken PNS (2007). Cognition, emotion, and neurobiological development: Mediating the relation between maltreatment and aggression. Child Maltreatment.

[b25] Lucas CP, First MB (2003). The use of structured diagnostic interviews in clinical child psychiatric practice. Standardized evaluation in clinical practice.

[b26] Mak AS (1994). Parental neglect and overprotection as risk factors in delinquency. Australian Journal of Psychology.

[b27] McLoone P (2004). Carstairs scores for Scottish postcode sectors from the 2001 Census.

[b28] Nickell AD, Waudby CJ, Trull TJ (2002). Attachment, parental bonding and borderline personality disorder features in young adults. Journal of Personality Disorder.

[b29] O'Dougherty Wright M, Crawford E, Del Castillo D (2009). Childhood emotional maltreatment and later psychological distress among college students: The mediating role of maladaptive schemas. Child Abuse and Neglect.

[b30] ONS (2000). Standard occupational classification.

[b31] Patton GC, Coffey C, Posterino M, Carlin JB, Wolfe R (2001). Parental ‘affectionless control’ in adolescent depressive disorder. Social Psychiatry and Psychiatric Epidemiology.

[b32] Prevatt F (2003). The contribution of parenting practices in a risk and resiliency model of children's adjustment. British Journal of Developmental Psychology.

[b33] Rutter M, Kim-Cohen J, Maughan B (2006). Continuities and discontinuities in psychopathology between childhood and adult life. Journal of Child Psychology and Psychiatry.

[b34] Teicher MH, Andersen SL, Polcari A, Anderson CM, Navalta CP, Kim DM (2003). The neurobiological consequences of early stress and childhood maltreatment. Neuroscience and Biobehavioral Reviews.

[b35] Teicher MH, Dumont NL, Ito Y, Vaituzis C, Giedd JN, Anderson SL (2004). Childhood neglect is associated with reduced corpus callosum area. Biological Psychiatry.

[b36] Trevarthen C, Aitken KJ (2001). Infant intersubjectivity: Research, theory, and clinical applications. Journal of Child Psychology and Psychiatry.

[b37] Turgeon M, Nolin P (2004). Relationship between neglect and children's memory and verbal learning capacities. Revue quesbecoise de psychologie.

[b38] West P, Sweeting H, Barton DG, Lucas C (2003). Voice-DISC identified DSM-IV disorders among 15-year-olds in the West of Scotland. Journal of the American Academy of Child and Adolescent Psychiatry.

[b39] Wilhelm K, Niven H, Parker G, Hadzi-Pavlovic D (2004). The stability of the Parental Bonding Instrument over a 20 year period. Psychological Medicine.

[b40] Winnicott D (1965). The maturational process and the facilitative environment.

[b41] Yates T, Wekerle C (2009). The long-term consequences of childhood emotional maltreatment on development: (Mal)adaptation in adolescence and young adulthood. Child Abuse and Neglect.

[b42] Yoshida T, Taga C, Matsumoto Y, Fukui AK (2005). Paternal overprotection in obsessive–compulsive disorder and depression with obsessive traits. Psychiatry and Clinical Neurosciences.

