# Supplementary material for: Children's perceptions of parental emotional neglect and control and psychopathology
Source: J Child Psychol Psychiatry. 2011 Mar 25;52(8):889–97. doi: 10.1111/j.1469-7610.2011.02390.x (PMC3170712; doi:10.1111/j.1469-7610.2011.02390.x)
Supplement: Supplementary file 1 [file jcpp0052-0889-SD1.doc]

Low Care

Low

Control

High

Control

Optimal

parenting

Neglectful

parenting

Affectionless

control

Affectionate constraint

Latent Class 2: Moderate parenting

(Mostly cared and sometimes controlled)

Latent Class 1: Typical parenting

(Often cared and sometimes controlled)

Latent Class 4: Neglectful/Controlling parenting'

(Uncared and controlled)

[fignote]Note: Solid arrows indicate congruence between latent classes and conventional PBI parental styles and those derived from LCA, while shaded arrows indicate a lack of congruence.

High Care

[fc]**Figure 1** Relationship between conventional ‘parental bonding’ parental styles and styles derived through latent class analysis

Latent Class 3: Optimal parenting

(Always cared and not controlled)
